# Development of Hybrid Composite Utilizing Micro-Cellulose Fibers Extracted from Date Palm Rachis in the Najran Region

**DOI:** 10.3390/polym14214687

**Published:** 2022-11-03

**Authors:** Hassan Alshahrani, Basheer A. Alshammari, Ahmer Hussain Shah, Abdul Qadeer Dayo

**Affiliations:** 1 Department of Mechanical Engineering, College of Engineering, Najran University, Najran 11001, Saudi Arabia; 2 Materials Science Research Institute, King Abdulaziz City for Science and Technology, Riyadh 11442, Saudi Arabia; 3 Faculty of Engineering and Architecture, Balochistan University of Information Technology, Engineering and Management Sciences, Quetta 83700, Pakistan

**Keywords:** date palm fibers, hybrid composites, mechanical properties, thermomechanical properties, thermal properties

## Abstract

Environmental effects can be reduced by using renewable resources in various applications. The date palm fibers (DPF) used in this study were extracted from waste date ranches of the Najran region by retting and manual peeling processes. The biocomposites were developed by reinforcing the silane-treated DPF (SDPF) at different wt.% in eugenol phthalonitrile (EPN) and difunctional benzoxazine (BA-a) copolymer. The impact strength, tensile, flexural, and dynamic mechanical properties and thermogravimetric analysis were evaluated to understand the mechanical, thermomechanical, and thermal properties. Results confirmed that 30 wt.% SDPF-reinforced poly (EPN/BA-a) composites produced the highest mechanical and thermomechanical properties, and were considered optimized SDPF reinforcement. Furthermore, hybrid composites with 30 wt.% SDPF and 15 wt.% silane-treated glass fibers (SGF) reinforcement having different lamination sequences were also studied. The lamination sequences showed a significant impact on the mechanical and thermomechanical properties, as properties were further enhanced by adding a core layer of SGF in hybrid composites. However, the thermal properties of SDPF/SGF laminates were higher than SDPF biocomposites, but the SGF lamination sequence did not produce any impact. According to the limiting oxygen and heat resistance indexes, the developed SDPF/SGF laminates are self-extinguishing materials and can be used in temperature-tolerant applications up to 230 °C.

## 1. Introduction

Evergreen date palm trees (*Phoenix dactylifera* L.) are widespread in the Middle East and the Arabian Peninsula due to the suitable climate. The date palm can easily survive an arid environment and provide enormous resources for nomadic tribes and local populations. The date palm tree requires special care and has a 40–50 year average life [1]. Worldwide, there are approximately 105 million palm trees covering an area of 800,000 ha (FAO 2020). Based on the National Center Report for palm and dates, the total number of date palm trees in the Najran region is around 385,623 trees. Every year, approximately 40 kg of biomass from a single date palm tree is generated from offshoot, dead, or defective fronds. The generated waste is burned or landfilled, which causes environmental pollution [2]. Date palm waste is an excellent biomass source, as they have a low moisture content. Each year, Saudi Arabia generates over 345,000 tons of date palm waste: dry leaves, stems, pits/seeds, etc. [3]. During the past few decades, the demand for date fruit has increased due to the cultural and traditional implications and increasing populations in the region and other Islamic countries. Thus, the cultivation of this ancient crop in the Middle East has been dramatically increased and is expected to continue. As a result, the generation of date palm waste is expected to increase in the future [4].

The physiochemical, thermal, and biochemical techniques have been applied for the sustainable utilization of date palm waste. The date palm waste is fibrous and primarily composed of cellulose that has been lignin-embedded. Moreover, the date palm fibers (DPF) have hollow channels due to the presence of lumen. The chemical composition of biomass fibers shows the effects on various characteristics such as weather resistance, degradation, fungi attack, etc. [5]. The date leaves, rachis, and stems are constituents of cellulose (55–75%), lignin (≈15–30%), ash (≈2–10%), and extractive (≈5–8%) [5].

The enforcement of inflexible environmental legislation for the composite industry has changed the momentum in favor of finding out environment-friendly reinforcements and resin systems by maintaining performance similar to their synthetic counterparts. Extensive research is being carried out on possible replacements for industrial fibers. In this context, the use of DPF extracted from biomass waste has many advantages compared to industrial fibers, such as carbon or glass fibers (GF). The advantages include ease and economical availability, as they are renewable resources and consume a lower amount of energy during the manufacturing process [6]. The use of extracted micro or nano DPF will have positive environmental and economical effects. Various studies exist in the literature on DPF reinforced composite, with low-density polyethylene [5,7], starch [8,9], polypropylene [10,11], polyester [12], phenolic [13], and epoxy [14,15]. However, the effects of DPF reinforcement in high performance thermosets are not studied.

Hybrid composites have more than single fiber materials in the same resin matrix. Hybrid composites offer distinguishing properties that may or may not be present in original elements. From the studies, it has been confirmed that the interplay hybridization with synthetic fibers is a promising solution to overcome drawbacks that reduce the outdoor applications of natural fiber-reinforced composite materials [16]. Several studies have been reported on the mechanical and thermomechanical properties of hybrid composites made with bio-based and synthetic fibers. Wang and coworkers evaluated the flax/GF sandwich structured hybrid composites made with epoxy resin [17]. They stipulated that the flax fibers as core and GF at the outer surface formation have the best thermomechanical and mechanical properties. Kumar et al. [18] prepared brake pads with alkali-treated DPF and compared the properties with traditional Kevlar-based brake pads. It was concluded that the hybrid brake pads developed with 5 wt.% alkali-treated DPF and 45 wt.% Kevlar fiber produced the best friction results. One most promising composite structure is a sandwich structure that can enable fibers to maintain their level and trap air in micro pockets, thus providing superior insulating properties along with lower weight and higher strength. Alarifi studied the effects of the DPF collected from leaves, branches, and core-shell on the prepared composites [19]. The epoxy date palm leaf composites had the best mechanical performance among the examined composites.

Phthalonitrile (PN) resins synthesized from petroleum-based phenolate salts and 4-nitrophthalonitrile have exceptional physical properties. However, high melting points (200–250 °C), small processing windows, and fluctuating prices due to crude oil-based raw materials are major concerns for the operational cost [20]. An easy and promising alternative to crude oil-based monomers is to synthesize bio-based monomers by maintaining their performance. Several bio-based PN monomers like guaiacol [21], cardanol [22], eugenol (EPN) [21], and hydroxymethylated eugenol-based [23] PN monomers with cost-effective fiber reinforced polymer composites fabrication process properties are reported in the literature [21,22]. However, the high curing temperature and long curing time are major drawbacks of these monomers. An easy and economical way to overcome the mentioned shortcoming is blending/copolymerization with new high-performance thermosets, for instance, novolac [24], epoxies [25], and benzoxazine [26]. Recently, the EPN and difunctional benzoxazine monomer (Bisphenol A-amine; BA-a) were copolymerized, and results established that the complete curing can be achieved at 240 °C in comparison to the pristine poly (EPN) which needs to be cured at 350 °C [21]. Moreover, the poly(BA-a/EPN) copolymer had higher glass transition temperature (*T_g_*), stiffness, and thermal stabilities in contrast to the virgin poly(BA-a) matrix [27].

The current study focuses on the amalgamation using the BA-a/EPN copolymer as a matrix to develop a composite laminate with silane-treated DPF obtained from biomass waste and hybrid composites with silane-treated glass fiber mat (SGF) by the compression molding process. The prepared hybrid laminates are intended to be applied in exigent applications for instance used as structural parts in the marine industry. The mechanical, thermomechanical, and thermal properties of biobased SDPF and SDPF/SGF hybrid composites were vigilantly scrutinized.

## 2. Materials and Methods

### 2.1. Materials

For the current study, Jiangxi Huacui Advanced Materials Co., Ltd. (Jiujiang, China) supplied the BA-a monomer. Aladdin Reagents Co. Ltd. (Shanghai, China) delivered eugenol (>99.0%), 4-nitrophthalonitrile (99.5%), and 3-glycidyloxypropyltrimethoxy silane agent (GPTM). GF mats with a plain-woven pattern, 200 gsm, and 2.45 g/cm^3^ density were procured from Beijing Chuanjing Co., Ltd. (Beijing, China). All AR-grade solvents and reagents used were supplied by Aladdin Reagents Co., Ltd. (Shanghai, China).

### 2.2. EPN Monomer

**Scheme 1:**
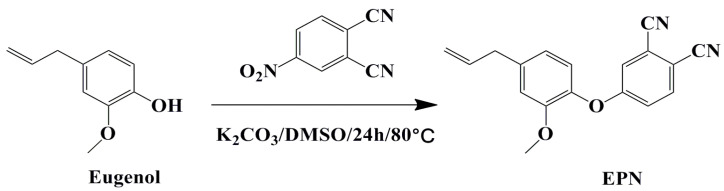
Synthesis route for EPN monomer.

The EPN monomer synthesis was carried out in the potassium carbonate presence by reacting eugenol and 4-nitrophthlonitrile, according to the reaction shown in Scheme 1, as suggested in prior work [21].

### 2.3. Fiber Isolation

The waste date palm ranches (leaf stem) were collected from a local farm in Najran, Saudi Arabia. The plant has an average of 15–25 ranches of 4–6 feet in length. The ranches were shredded to 6–10 inch in length before any physical processing or chemical treatment. The sand, dust, and other deposits attached to the ranches’ surface were removed by washing several times with tap water, and were then immersed in warm water (50 °C) for 24 h. Retting and manual peeling processes were employed to extract the DPF [28]. The extracted DPF was ground in an ultra-fine grinder to achieve the micron size. The DPF passed from 150 µm aperture size (100 mesh screen) were overnight soaked at ambient conditions in the acetone solution for the removal of containments from the surface and rinsed with distilled water. Later, the DPF were filtered and overnight vacuum dried at 110 °C. Subsequently, the obtained DPF micro-fibers were stored in air-tight bags and subjected to silane treatments.

### 2.4. Silane Treatment of DPF and GF

The DPF surface contains lignin, pectin, and waxy substances, which must be removed to obtain cellulose. The alkali treatment is considered an economical and easy way to achieve lignin, pectin, and waxy substance-free fibers. However, the silane treatment method gives better results as compared to alkali treatment [29].

**Scheme 2:**
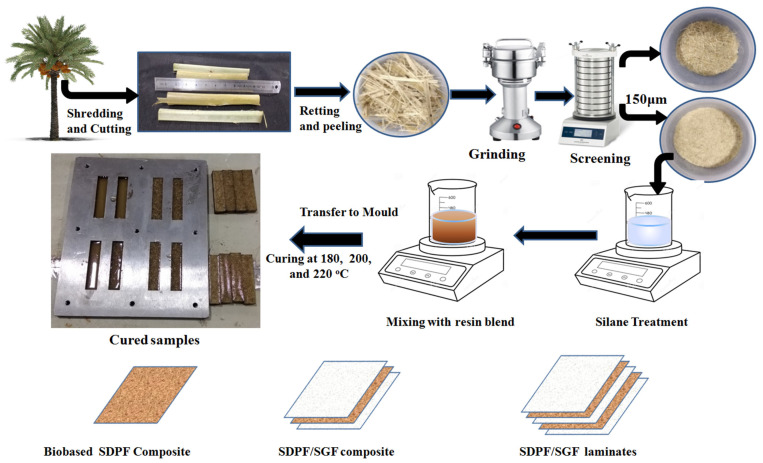
Procedure followed for SDPF fiber extraction and composites preparation.

The GPTM silane coupling agent solution was prepared by an amalgamation of 8 g GPTM in 100 mL ethanol and 100 mL deionized water. The DPF were magnetically stirred in the solution at ambient conditions for 6 h. Later on, DPF were filtered and the coupling agents’ adverse impact was reduced by rinsing with ethanol and deionized water solution in 1:1. The silane-treated DPF (SDPF) were vacuum dried at 85 °C for 6 h and stocked in an air-tight bag. The same procedure was followed to obtain the silane-treated GF (SGF) mat. Scheme 2 represents the procedure followed for the fiber extraction and composites preparation.

### 2.5. Processing of SDPF Composites and SDPF/SGF Hybrid Composites

The isothermal compression molding method was employed in the SDPF/copolymer and SDPF/SGF hybrid composites. The copolymer matrix was prepared by blending BA-a and EPN monomers in 6:4 for all samples. Different wt.% SDPF reinforced composites were obtained by following the composition mentioned in Table 1.

The monomers were melted and homogenized on a hot plate, and the SDPF weight amount as per the given composition mentioned in Table 1 was well mixed in the monomer blend. Later on, the steel molds with test sample dimensions were filled with the mixture. The mixture was degassed in a vacuum oven at 140 °C for 4 h for voids revoking from the cured composite. The test specimens were cured at 160, 180, 200, and 220 °C for 2 h at each temperature on 12 MPa in an isothermal compression molding machine. The SDPF/SGF hybrid composites with copolymer resins were also prepared following the earlier mentioned procedure, only SGF mats were soaked in the monomer blends, and the stacking sequence of the sandwich hybrid composites is shown in Table 1. The following equation was used to determine the total fiber volume fraction (*V_f_*) [30].

(1) $$V_{f}=\frac{\left(w_{D}/\rho_{D})+(w_{G}/\rho_{G}\right)}{\left(w_{D}/\rho_{D})+(w_{G}/\rho_{G})+(w_{M}/\rho_{M}\right)}$$

where *w* presents the weight in fraction and *ρ* presets the density. The subscripts *D*, *G*, and *M* represent the respective values of the SDPF, SGF, and copolymer matrix.

### 2.6. Characterization

#### 2.6.1. FTIR

A Perkin Elmer Spectrum 100 spectrometer (Waltham, MA, USA) was used for the confirmation of the silane treatment on the fibers’ surface; a thin sample film with KBr was cast and evaluated in 4000–500 cm^−1^ region to obtain the Fourier transform infrared (FTIR) spectrum of fibers.

#### 2.6.2. Mechanical Properties

For the analysis of the mechanical properties of the developed composites, tensile, flexural, and impact strength properties were evaluated. An impact resistance (IT503 Tinius-Olsen, Shanghai, China) was used to estimate the impact strength, while the ASTM D256-2010 standard was followed during testing. The Instron 5569 instrument (Norwood, MA, USA) was used to evaluate the tensile and flexural (three-point bending) properties at 1 and 2 mm/min^−1^ crosshead speed, respectively, of 50 × 10 × 2 mm^3^ rectangular samples. For a single code specimen, at least four replicas were tested and the average value is reported.

#### 2.6.3. Dynamic Mechanical Analysis

The thermomechanical properties of developed biobased composites, hybrid laminates, and pristine copolymer matrix samples with a dimension of 30 × 5 × 2 mm^3^ were evaluated on a dynamic mechanical analyzer (DMA), Q800, TA Instruments (New Castle, DE, USA), from 30 to 325 °C at a 5 °C/min heating ramp and 1 Hz frequency.

#### 2.6.4. Thermal Stabilities

A TA Instruments Q50 (New Castle, DE, USA), was employed to determine the thermal stabilities from 50 to 750 °C in N_2_ purging (50 mL/min) of the developed biobased composites, hybrid laminates, and pristine matrix samples at 20 °C/min heating rate.

#### 2.6.5. Water Absorption

ASTM D570-98 standard was followed for the estimation of water absorption of the copolymer matrix, SDPF composite, and SDPF/SGF hybrid composites. Five rectangular specimens for a single code were studied and water absorption was calculated from the following formula.

(2) $$W_{u}=\frac{\left(W_{t}-W_{o}\right)}{W_{o}}\;100\%$$

Here *W_u_* represents the water uptake in percentage; while *W_t_* is the weight of the sample at time *t* and *W_o_* represent the weight of the sample before initiating the test. 

## 3. Results and Discussion

### 3.1. Effects of Silane Treatment on Fibers

The silane coupling agent increases the interfacial bonding between the natural fiber and the matrix by reducing the cellulose hydroxyl groups of natural fiber. The silane-treated fibers form covalent bonding between the fibers and matrix, this also prevents the swelling of fibers on exposure to moisture [31]. The chemical structure and thermal stabilities before and after the fibers’ surface modification were evaluated by employing the FTIR and TGA, respectively.

**Figure 1:**
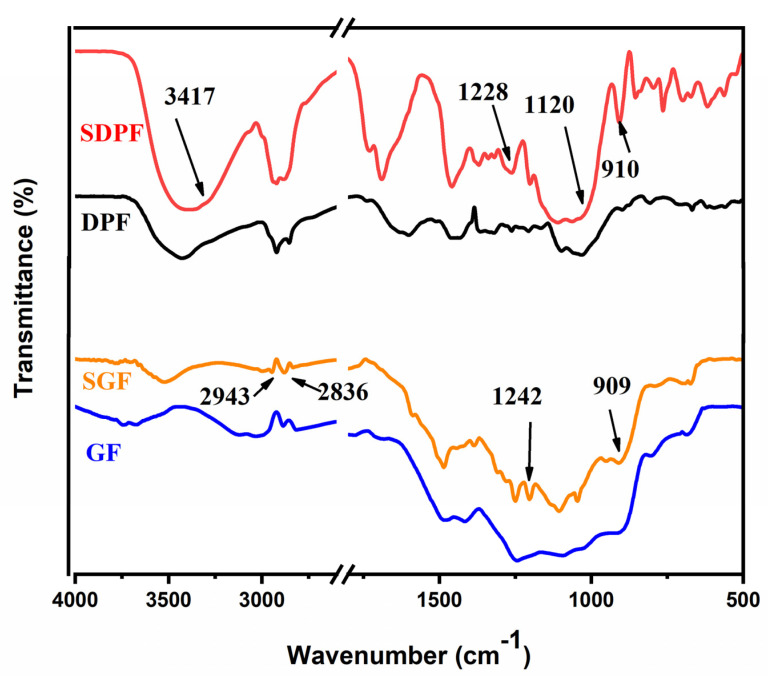
FTIR spectra of untreated and silane-treated fibers.

From the produced FTIR curves in Figure 1, it can be easily observed that the silane treatment has effectively modified the fiber composition. The lignin (C=O in the aromatic ring, at 1228 cm^−1^), pectin and wax (1735 cm^−1^), and hemicellulose (C=O, at 1648 and 1729 cm^−1^) were reduced [32]. Moreover, the silane treatment increased the intensity of the cellulose -OH stretching peak at 3417 cm^−1^. This suggests that the silane coupling agent enhanced the -OH concentration, and reduced the fibers’ hydrophilic nature; these -OH groups will enhance the exposure to the matrix for the interaction. Furthermore, the silane-DPF chemical reaction was confirmed by the polysiloxane network in the SDPF by the formation of O-Si-O bonds (1120 cm^−1^) [33]. 

Considering the FTIR spectra of GF, the untreated GF has very few peaks confirming lower number of specific functional groups. This suggests that the GF will have lower physical and chemical interactions with the matrix [34]. However, the SGF surface produced several specific functional groups at 1242, 841, 909, 2943, and 2836 cm^−1^, representing the existence of Si-O-Si, Si-C, epoxy, CH_2_, and CH_3_, respectively. Since the silane groups can easily react with the generated hydroxyl groups during the resin curing process, SDPF and SGF fibers will have a strong chemical bond with the matrix [35].

**Figure 2:**
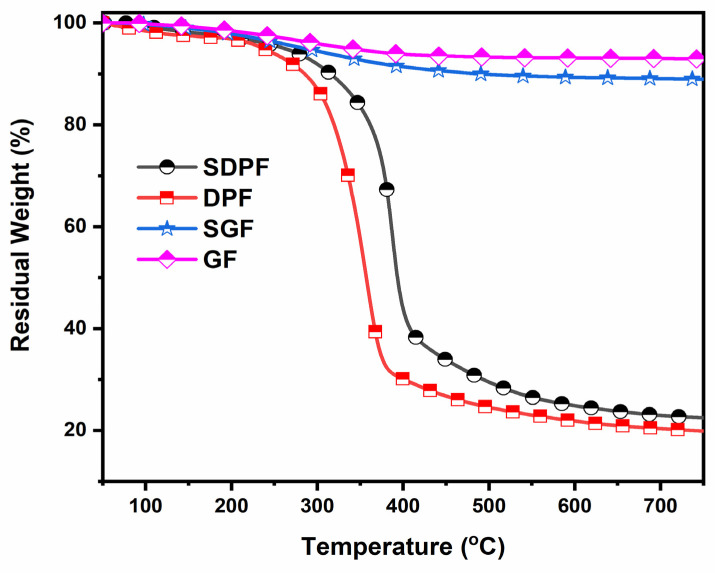
TGA thermograms in N_2_ environment of untreated and silane-treated fibers.

The BA-a/EPN copolymer is cured at a higher temperature range as compared to traditional thermosets like epoxy or phenolic resins. During the curing process, the natural fibers will be exposed to the higher temperature, thus understanding the fibers’ behavior at elevated temperatures (thermal stability) is an important parameter. The thermal stabilities of silane-treated DPF and GF were studied and the produced TGA results are illustrated in Figure 2. The extracted data from TGA spectra are summarized in Table 2.

The 5 and 10 wt.% loss temperatures (*T*_5_ and *T*_10_) of the untreated DPF were observed at 236 and 282 °C, respectively. Moreover, a very diminutive weight loss nearby 100 °C confirmed that the moisture was sufficiently removed and DPF was properly stored in airtight bags. After the silane treatment, the *T*_5_ and *T*_10_ values were increased to 261 and 315 °C, respectively. Furthermore, a rise was recorded in the residual mass amount at higher temperatures known as char yield value (*Y_c_* at 750 °C). The introduction of a higher alkyl chain length and silyl part of GPTMS not only increased the thermal stability of the fiber but also improved the hydrophobicity of SDPF [36].

The GF is composed of highly thermally stable compounds i.e., SiO_2_, Al_2_O_3_, MgO, CaO, and ZnO [37,38], therefore the GF has excellent thermal stability in comparison to DPF. For instance, *T*_5_ and *Y_c_* for the as-received GF were about 337 °C and 92.9%, respectively. The *T*_5_ and *Y_c_* values recorded for silane-treated GF were slightly lower in comparison to the corresponding values of as-received GF and recorded as 285 °C and 88.92%, respectively. This can be attributed to the attached organic compounds after the silane treatment, as they can be easily degraded at lower temperature [39]. The FTIR and TGA results confirmed the effective treatment of silane coupling agents on DPF and GF.

### 3.2. Mechanical Properties

#### 3.2.1. Impact Properties

**Figure 3:**
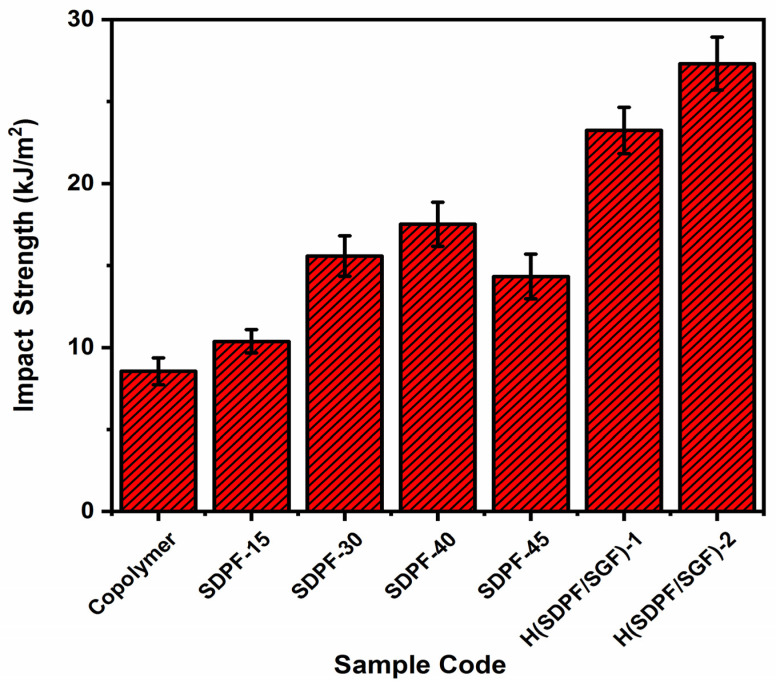
Comparison of impact strength of SDPF-reinforced biocomposites and SDPF/SGF hybrid composites.

Figure 3 presents the comparison of impact properties of neat copolymer resin and SDPF-reinforced composites. The impact strength value for the neat copolymer matrix was estimated to be about 8.55 ± 0.82 kJ/m^2^. Initially, on lower SDPF wt.% reinforcement (15 wt.%), a moderate increase (21.5%) was observed in the impact strength value. On further increase in the SDPF wt.%, a huge rise of 82.3% in the impact strength of SDPF reinforced composites was observed. However, 45 wt.% SDPF reinforced composite showed a slight decline in the impact strength values (8% lower as compared to the SDPF-30 sample). This suggests a gradual rise in the impact strength and the SDPF wt.%. Moreover, the impact strength of the 40 wt.% SDPF reinforced composite (SDPF-40) sample was 105% higher than that of the neat matrix. The decline in the impact strength as in the SDPF-45 sample case can be credited to poor fiber-matrix adhesion due to a lower amount of matrix volume, this agglomerates the fibers and decreases the energy needed for fiber pull-out [40].

The effect of SGF hybrid composites with optimum SDPF fraction and various SGF sequences on the impact strength is also presented in Figure 3. The SGF hybrid composites produced a further rise of 7.65 kJ/m^2^ in the impact strength value in contrast to the optimum 30 wt.% SDPF reinforced composites. Additionally, the impact strength values are greatly affected by the modification in layup sequences while maintaining the same blending ratio, as in the case of the H(SDPF/SGF)-1 and H(SDPF/SGF-2) specimens. The H(SDPF/SGF)-2 specimen has a rise of 4.08 kJ/m^2^ in impact strength compared to the H(SDPF/SGF)-1specimen. A thin GF mat layer in the center of the hybrid composites increases the impact resistance compared to the only thick layers outside. 

#### 3.2.2. Tensile Properties

**Figure 4:**
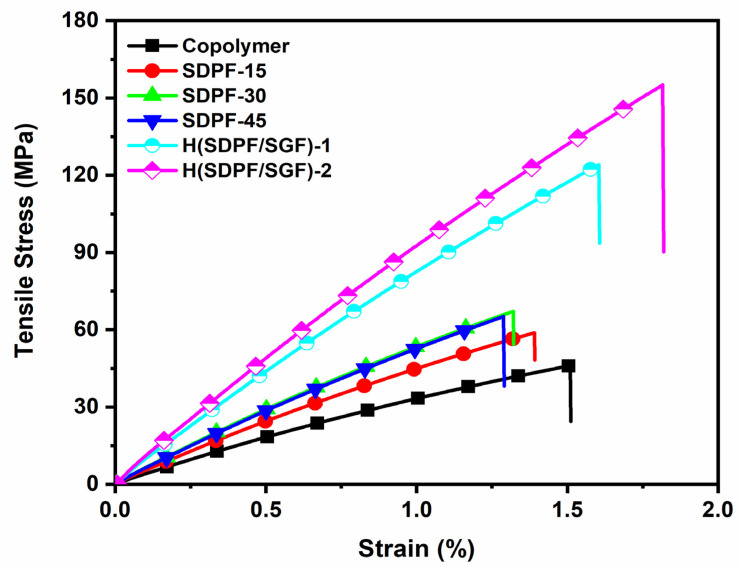
Tensile stress-strain curves of SDPF and SDPF/SGF hybrid composites.

**Figure 5:**
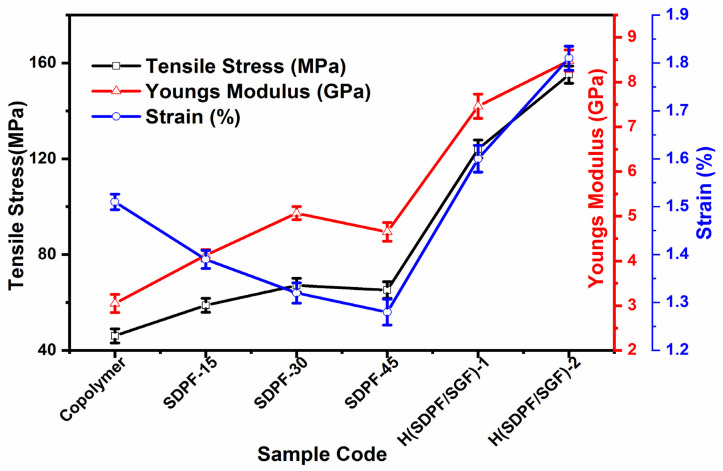
A summary of tensile stress-strain curves of SDPF and SDPF/SGF hybrid composites.

All tensile stress-strain curves showed catastrophic failure with linear behavior up to a certain point. Obtained curves of the pristine copolymerized matrix, SDPF reinforced biocomposites, and SDPF/SGF hybrid composites are plotted in Figure 4. For a better understanding of the relationship between tensile properties, a summary of obtained values from tensile stress-strain curves is plotted in Figure 5.

The lowest tensile strength (46.08 ± 2.98 MPa) and Young’s modulus (3.05 ± 0.20 GPa) were observed for the pristine copolymer matrix, whereas it also had a higher strain to failure among SDPF-reinforced composites. The highest tensile strength and Young’s modulus values among SDPF reinforced composites were recorded on 30 wt.% SDPF reinforcement, the respective values were recorded as 67.15 ± 2.78 MPa and 5.07 ± 0.14 GPa. On further SDPF reinforcement (45 wt.%), the tensile strength and modulus values were slightly reduced due to the agglomeration which reduced the interfacial bonding between fiber and copolymer resin [41] and recorded as 65.17 ± 3.45 MPa and 4.65 ± 0.21 GPa, respectively.

The DPF microfibers were used as reinforcing materials, which have a lower elongation at break. Thus, the 45 wt.% SDPF reinforced composites showed nearly 15.3% and 29.3% lower elongation at break as compared to pristine matrix and SDPF/SGF hybrid composites, respectively, because GFs mat has higher elongation as compared to micro SDPF. Furthermore, the SDPF/SGF hybrid composites showed a rapid rise in stress to the maximum value, followed by fracture failure, with the failure strain 37.1% higher than the SDPF reinforced composites. Thus, SDPF/SGF hybrid composites increase the ductility along with a rise in tensile strength and Young’s modulus. The GF have much higher strength values as compared to the DPF. The lamination methods have a positive effect on the tensile strength and Young’s modulus of hybrid composites. The inner layer SGF hybrid composite showed an additional rise of 25% and 13% in the tensile strength and Young’s modulus values, respectively, as compared to the recorded values for a traditional hybrid composite having SGF on the outer layers. Furthermore, in our earlier study, we evaluated chopped SGF composites with poly(BA-a/EPN) copolymer having the same composition [42]. The tensile strength and Young’s modulus of the SDPF/SGF sandwich structure were nearly 40 MPa and 2.5 GPa higher than the 40 wt.% chopped SGF reinforced composite.

#### 3.2.3. Flexural Properties

**Figure 6:**
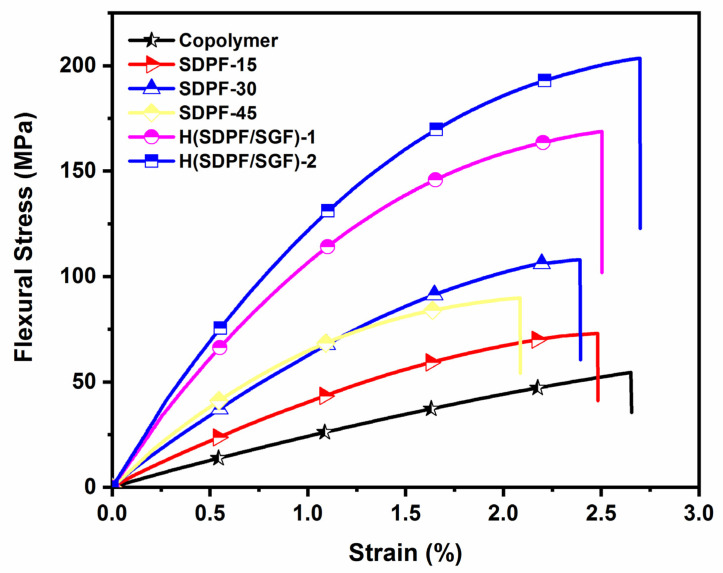
Three-point bending stress-strain curves of SDPF and SDPF/SGF hybrid composites.

The flexural stress-strain curves of the biobased SDPF and hybrid SDPF/SGF composites are presented in Figure 6. A summary of obtained values from bending stress-strain curves and a comparison with other studies is presented in Table 3.

The pristine matrix showed the lowest flexural strength (53.42 ± 3.69 MPa) and flexural modulus (2.03 ± 0.32 GPa) due to the brittle nature of the matrix. The SDPF reinforcement in the copolymer improved the flexural strength and modulus and a decline was recorded in the bending strain values.

The hybridization with GFs had a positive effect on the SDPF composites. The bending strain at break of the GF mat was much better than the SDPF, and the same was replicated in flexural stress-strain curves. The SDPF/SGF hybrid composites showed higher bending strain at break as compared to the pristine matrix and biobased SDPF composites. However, the stress-strain curve of the SDPF/SGF hybrid composite showed a swift rise in strength and a sudden reduction after reaching the maximum value. Moreover, the results confirmed that the hybrid laminates have better values in comparison to SDPF composites. Furthermore, from the plots it can be observed that H(SDPF/SGF)-1 and H(SDPF/SGF)-2 specimens have the same blending ratio, however, the variation in layup sequences significantly affected the flexural strength and modulus. The addition of the intermediate layer further added 34.84 MPa and 1.45 GPa in flexural strength and modulus values, respectively.

Furthermore, the flexural properties of the hybrid composites were significantly higher than the various studied composites; i.e., 40 wt.% chopped GF reinforced in BA-a/EPN copolymer [42], 20 wt.% Kevlar Fiber reinforced in poly(BA-a) [43], and flax/basalt fibre laminates with epoxy resin [44]. However, the flexural strength and modulus were significantly lower than the flax/GF hybrid epoxy composites [17]. This can be attributed to the usage of the flax fiber mats in earlier investigations, whereas the laminates in the current study were made using micro SDPF fibers. These findings imply that the investigated composites exhibit excellent properties and are suitable for usage in the demanding application in structural engineering.

### 3.3. Thermomechanical Properties

**Figure 7:**
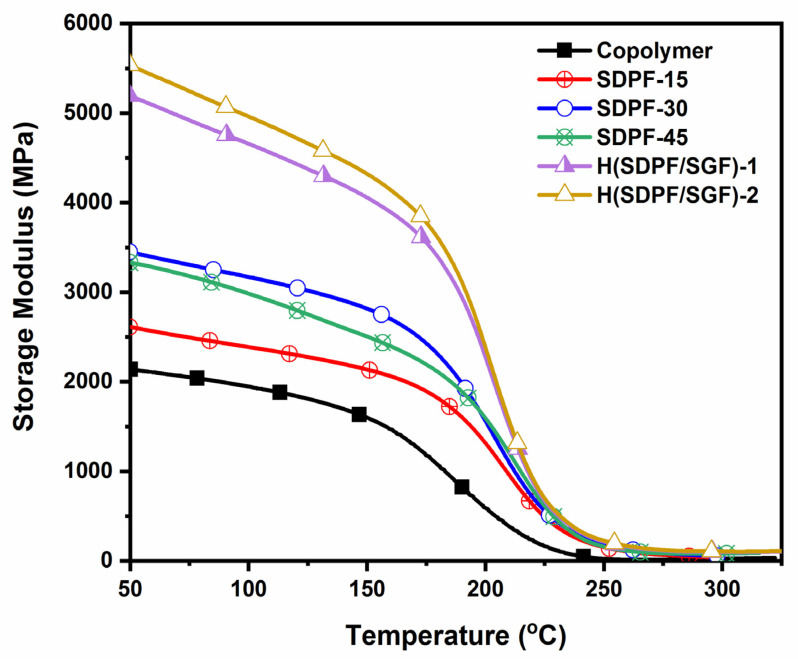
The storage modulus curves of SDPF biocomposites and SDPF/SGF hybrid laminates with poly(BA-a/EPN) copolymer as a matrix.

During the selection of polymer-based materials for high-temperature applications, the thermomechanical properties are significantly considered. The DMA is considered an effective way to determine the thermomechanical properties of polymer-based materials, such as the ability to retain stored energy. Thermomechanical properties of polymer-based composites, exclusively depend on the reinforced material’s nature and morphology, and generated interface between reinforcement and matrix [46]. Figure 7 presents the storage modulus curves versus temperature of SDPF composites and SDPF/GF hybrid composite with poly(BA-a/EPN) copolymer as a matrix, and the extracted data from the DMA spectra are summarized in Table 4.

From the spectra plotted in the figure, we can easily scrutinize the enhancement in the storage modulus (*E’*) due to the SDPF reinforcement till 30 wt.%. This can be attributed to the stiffness imposed by the fibers primarily by the strength of the intermolecular forces and stronger adhesion to the polymer chains at the fiber/matrix interface. The SDPF-45 sample having 45 wt.% SDPF reinforcement showed nearly 4% lower *E’* value as compared to SDPF-30 sample. This observation is in good agreement with the earlier observed mechanical properties. On a rise in the temperature, a sharp fall in *E’* value was observed nearby the *T_g_* due to the diffusion motion of the main chain segments known as Brownian movements [47]. Moreover, the decline in *E’* is very high for the neat copolymer matrix in comparison to biobased or hybrid composites, due to the escalation in the mechanical restraint and hydrodynamic effects. The highest *E’* value was recorded for the H(SDPF/SGF)-2 hybrid laminate.

The reinforcing effect can be evaluated by computing the effectiveness coefficient (*C*), defined as the ratio between the *E’* values in glassy and rubbery regions of the composites in relation to the pristine matrix, and represented by the following equation [48].

(3) $$C=\frac{{\left(\;E_{g}^{\prime }/E_{r}^{\prime }\right)}_{Composite}}{{\left(\;E_{g}^{\prime }/E_{r}^{\prime }\right)}_{Resin}}$$

The coefficient *C* was computed at three temperatures in the rubbery region (175, 200, and 225 °C), while the *E’* value in the glassy region was kept constant at 50 °C mostly known as stiffness. 

From the analysis of *C* values for the SDPF-reinforced biobased composites, it can be concluded that the *C* value decreases as the SDPF content increases till the optimized loading. The lower *C* value signifies better effectiveness and advocates the higher stress transfer between fiber and matrix. On 45 wt.% reinforcement either for biobased SDPF composites or hybrid (SDPF/SGF) laminates. The *C* values do not change, due to the possible agglomeration caused by the increased volume fraction. Similar behavior is already reported for pineapple leaf/GF [49] and ramie fiber/GF hybrid polyester composites [50].

**Figure 8:**
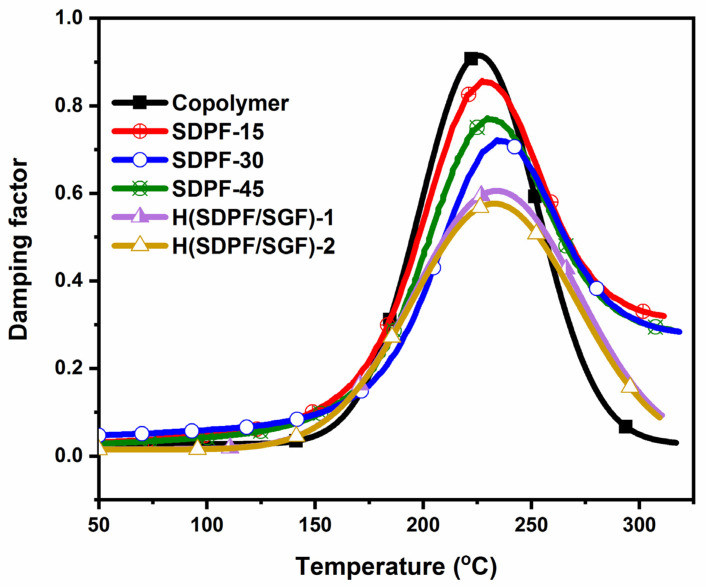
The damping factor spectra of SDPF biocomposites and SDPF/SGF hybrid laminates.

The reinforcements, either fibers or particles, show significant impacts on the damping factor (*tan delta*) spectra of the obtained composites. From the *tan delta* peak height and *T_g_* values, the possible filler-matrix adhesion can be estimated. The weak filler-matrix adhesion produces a higher value for *tan delta* peak height and a lower *T_g_* value. While stronger filler-matrix adhesion produces vice versa results due to the limited mobility of the polymer chains [46]. Figure 8 presents the damping factor curves of SDPF composites and SDPF/GF hybrid composites.

From the *tan delta* curves, it can be observed that the curves’ peak height decreased and shifted towards a higher temperature as the SDPF fiber content was gradually raised from 0 to 30 wt.%. The 45 wt.% SDPF reinforced biocomposites showed a slightly higher damping factor peak. The lower peak height indicates good interfacial adhesion [51]. Moreover, the curve width also becomes broader upon fiber addition. The H(SDPF/SGF)-2 hybrid laminates showed the highest *T_g_* (233 °C). The *T_g_* value of hybrid laminates was slightly lower than the SDPF-30 biobased composites. Similar observations were recorded for empty fruit bunch/jute hybrid epoxy laminates [52], and pineapple/glass hybrid polyester laminates [49]. Researchers dedicated this decline in *T_g_* due to lower fiber/matrix interaction on the lower volume of the matrix.

### 3.4. Thermal Properties

**Figure 9:**
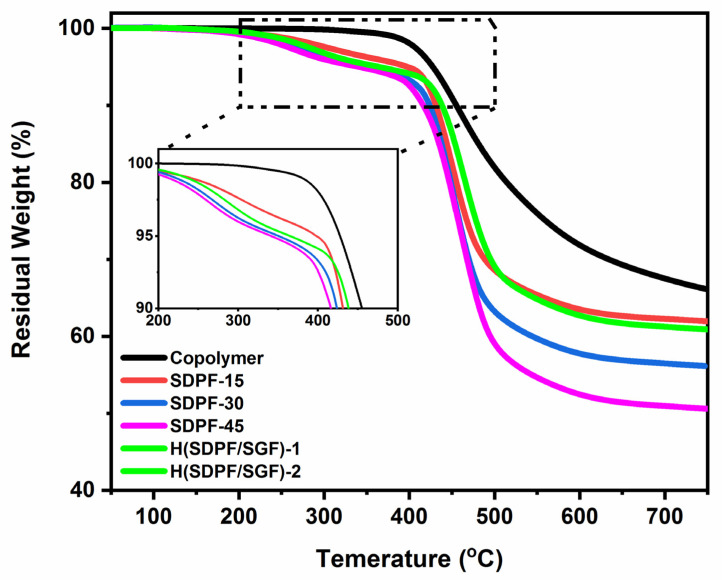
Thermal stability spectra of SDPF biocomposites and SDPF/SGF hybrid laminates in N_2_ atmosphere.

Thermal stability behavior of the developed biobased SDPF and SDPF/SGF hybrid composite laminates were scrutinized through TGA in an N_2_ environment; produced spectra are illustrated in Figure 9, and a summary of the TGA data is presented in Table 5.

From the TGA spectra for pristine copolymer matrix, biobased and hybrid composites, without any doubt, it can be observed that all composites and laminates have lower thermal stabilities than the matrix. Moreover, the copolymer matrix showed higher values for initial degradation (*T*_5_ and *T*_10_) and *Y_c_.* The recorded values were read as 427.6 °C, 455.2 °C, and 66.1%, respectively. These values were achieved due to the much better cross-linking of PN and polybenzoxazine resin. The thermal stability of biobased composites was gradually reduced as the SDPF reinforcement was raised from 0 to 45 wt.%, and a decline of 86 °C, 38.7 °C, and 15.5% were recorded in *T*_5_, *T*_10_, and *Y_c_* values, respectively. The SDPF are lignocellulosic fibers; on exposure to a higher temperature; the hemicellulose, lignin, and pectin were decomposed [53] and had lower thermal stability compared to the matrix. These observations trend is in good agreement with the thermals stability of DPF/epoxy composites recorded by Gheith and coworkers [54]. However, the DPF/epoxy composites showed a 25 wt.% decline at around 300 °C. In the current study, the improved thermal stabilities as compared to DPF/epoxy composites can be dedicated to the formed isoindoline and triazine structure during copolymer curing [55].

Furthermore, the SDPF/SGF hybrid composites showed a rise in *T*_5_, *T*_10_, *Y_c_*, *HRI*, and *LOI* values in contrast to SDPF-30 and SDPF-45 specimens. These composites have 30 and 45 wt.% SDPF reinforcement, while the hybrid composites have 30 and 15 wt.% reinforcement of SDPF and SGF, respectively. Synthetic fibers such as SGF have higher thermal stability in comparison to lignocellulosic-based natural fibers [56]. Thus, the initial degradation and residue values were significantly raised and recorded as 364.8 °C, 438.3 °C, and 60.89 for *T*_5_, *T*_10_, and *Y_c_* values, respectively. The lamination sequence does not show any impact on the thermal stability as both samples have the same composition. These can be dedicated to the better dispersion of fibers and accuracy during sample curing.

Heat resistance and limiting oxygen indexes (*HRI* and *LOI*) are very important terms to identify the limitation of the composites’ application. The *HRI* identifies the physical heat (temperature) tolerance limitation, while the *LOI* value > 26 indicates the excellent self-extinguishing and flame retardancy properties of the materials. The following relationships were calculated for the estimation of *HRI* and *LOI*, respectively [57].

(4) $$HRI=0.49\left[T_{5}+0.6\left(T_{30}-T_{5}\right)\right]$$

(5) $$LOI=17.5+0.4\;Y_{c}$$

From the calculated values as summarized in Table 5, we can easily analyze that the pristine copolymer matrix has a very high value for *HRI* and *LOI*, confirming the high-temperature tolerance and excellent self-extinguishing properties of the copolymer. Moreover, after the reinforcement of SDPF, the *HRI* and *LOI* values sharply decrease due to the lower thermal stabilities of reinforced material. The lowest *HRI* and *LOI* value among the studied biobased composites were observed for the SDPF-45 specimen having 45 wt.% SDPF reinforcement and recorded values were read as 204.25 °C and 37.74%, respectively. This suggests that the biobased SDPF composite laminates can be used up to 200 °C and have excellent self-extinguishing properties. Furthermore, the hybrid SDPF/SGF composite laminates showed a rise in the *HRI* and *LOI* value, and values were calculated to be 230.26 °C and 41.85%, respectively. This can be easily attributed to the synthetic fiber reinforcement having higher thermal stabilities as compared to the SDPF fiber [58]. Moreover, the *HRI* value of the hybrid composite is nearly the same as reported for the 30 wt.% carbon fiber reinforced in (dicyanate ester/benzoxazine) copolymer by Zegaoui and coworkers [59]. These observations are in favor of the TGA discussed earlier.

### 3.5. Water Absorption

**Figure 10:**
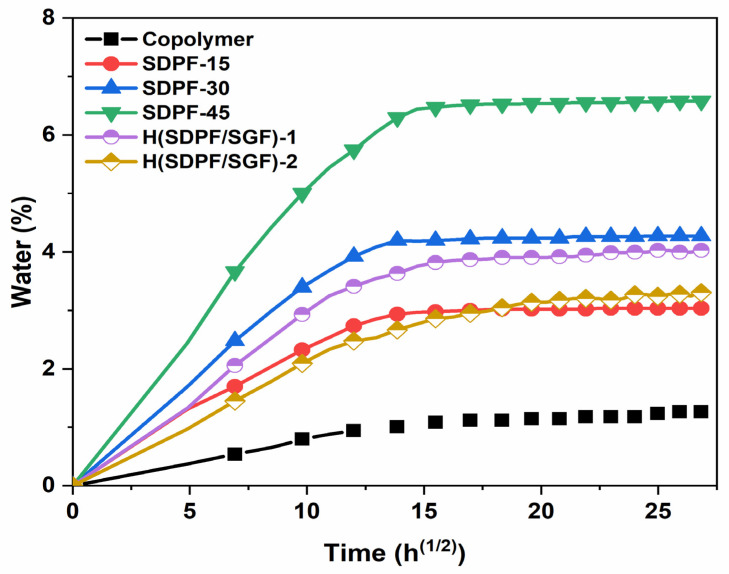
Water uptake behavior of SDPF and SDPF/SGF hybrid composites.

One of the critical challenges for natural fiber-reinforced composites is moisture susceptibility. The fiber-matrix interfacial interaction is weakened due to the absorbed water, and it ultimately reduces the mechanical properties[60]. The weight gain in percentage at room temperature as a function of the square root of time of SDPF biocomposites and SDPF/SGF hybrid composites is produced in Figure 10.

As expected, a gradual rise in the moisture absorption for SDPF biocomposites and SDPF/SGF hybrid composites continued till saturation was attained, however the moisture transport rate was reduced as the time of immersion was increased. The lowest moisture (1.26 ± 0.09%) was absorbed by the pristine matrix, whereas 45 wt.% SDPF reinforced composites absorb the highest moisture by 6.57 ± 0.12%. However, the lowest value (3.03 ± 0.11%) among the SDPF composites was recorded for the 15 wt.% SDPF, even after the saturation time. The SDPF/SGF hybrid composites also showed lower values compared to the SDPF-30 sample. Modest moisture absorption (4.02 ± 0.11%) was observed for H(SDPF/SGF)-1 sample having SGF mat on the outer side. However, the hybrid composites with an SGF mat on the outer side and core showed a slightly higher value (3.30 ± 0.1%) than the SDPF-15 sample, even though the sample contains 30 wt.% of SDPF. This confirms that moisture absorption can be reduced by an interlaminar layer of synthetic fiber in biocomposites.

The natural fibers such as DPF have -OH and contain amorphous regions which facilitate the water molecules’ diffusion and break the hydrogen bonds. Therefore, an intermolecular distance is created in the cellulose chains and consequently, the fibers are swollen [61].On the other hand, synthetic fibers like SGF do not absorb moisture from the surrounding environment or on water immersion [62]. Therefore, the affinity to absorb moisture by the hybrid laminates having SGF layers on the outer surface was sufficiently reduced. Similar observations were recorded by Cheng et al. in the durability study of carbon/flax fiber hybrid biocomposites with polypropylene matrix [40]. They concluded that the synthetic fibers sufficiently decline the water absorption, and the carbon/flax fibers hybrid composites having interlaminar carbon fiber mat layer obtained the lowest water absorption value (around 12.5%).

## 4. Conclusions

The reinforcement of SDPF fibers extracted from waste date palm ranches and the production of SDPF/SGF hybrid laminates in high performance poly (EPN/BA-a) thermosets were outlined in the current work. The biocomposites produced by 15–45 wt.% SDPF reinforcement showed that the mechanical properties were gradually enhanced and reduced after the optimized reinforcement of 30 wt.% SDPF. However, the thermal properties were significantly reduced due to the incorporation of the low thermally-stable SDPF compared to the copolymer matrix, as evidenced by the TGA results. For enhancement of the mechanical, thermomechanical, and thermal properties, SDPF/SGF hybrid laminate with 30 and 15 wt.% reinforcement of SDPF and SGF, respectively, and different lamination sequences were also developed. The developed SDPF/SGF hybrid laminates showed much better mechanical, thermomechanical, and thermal properties. The mechanical and thermomechanical properties were significantly enhanced by adding a core layer of SGF in hybrid laminate. Moreover, the lamination sequence does not show any impact on the thermal properties. From the *HRI* and *LOI* values, it was confirmed that the developed SDPF/SGF laminates have high-temperature tolerance (230 °C) and self-extinguishing properties.

## Figures and Tables

**Table 1:** Formulation and stacking sequence of biobased composites and hybrid laminates.

Specimen Code	BA-a [wt.%]	EPN [wt.%]	SDPF [wt.%]	SGF	*V_f_*	Stacking Sequence
Copolymer	60	40	-	-	0	Uniform
SDPF-15	51	34	15	-	0.185	Uniform
SDPF-30	42	28	30	-	0.355	Uniform
SDPF-45	33	22	45	-	0.512	Uniform
H(SDPF/SGF)-1	33	22	30	2 Layer (7.5 wt.% each layer)	0.458	□×□
H(SDPF/SGF)-2	33	22	30 (15 wt.% each layer)	3 Layer (5 wt.% each layer)	0.458	□×□×□

Note: × = SDPF, □ = SGF.

**Table 2:** TGA summary of untreated and silane-treated DPF and GF.

Sample	*T*_5_ [°C]	*T*_10_ [°C]	*Y_c_* at 750 °C [%]
Untreated DPF	236	282	19.88
SDPF	261	315	22.47
Untreated GF	337	-	92.91
SGF	285	421	88.92

**Table 3:** Comparison of flexural properties of the SDPF and SDPF/SGF reinforced hybrid composites with literature.

Sample Code	Flexural Properties			Reference
	Strength [MPa]	Modulus [GPa]	Strain [%]	
Copolymer	53.42 ± 3.69	2.03 ± 0.32	2.65 ± 0.07	Current Work
SDPF-15	73.03 ± 5.32	3.12 ± 0.25	2.48 ± 0.09	
SDPF-30	107.82 ± 4.37	4.58 ± 0.25	2.39 ± 0.05	
SDPF-45	89.81 ± 6.16	4.79 ± 0.54	2.08 ± 0.06	
H(SDPF/SGF)-1	168.71 ± 4.36	8.17 ± 0.35	2.51 ± 0.03	
H(SDPF/SGF)-2	203.55 ± 4.71	9.62 ± 0.32	2.69 ± 0.04	
Chopped GF-40 wt.% ^a^	148.64 ± 4.36	7.74 ± 0.216	1.38 ± 0.04	[42]
Kevlar fiber-20 wt. % ^b^	149.78 ± 7.4	6.89 ± 5.3	-	[43]
Flax/basalt fiber ^c^	144.8	8.27	-	[44]
DPF/kenaf fiber ^d^	56.24	4.77	-	[45]
Jute/basalt fiber ^d^	178.6 ± 2.9	10.60 ± 0.33	-	[16]
Flax/GF ^d^	390.19	13.56	-	[17]
Carbon cloth ^e^	756	119	0.9	[23]

^a^ Reinforced in poly(BA-a/EPN) copolymer in 6:4; ^b^ poly(BA-a) benzoxazine; ^c^ Vinyl ester; ^d^ Epoxy; ^e^ hydroxymethylated eugenol PN.

**Table 4:** Summary of stiffness, *tan delta,* and effectiveness coefficient (*C*) from DMA spectra of SDPF biocomposites and SDPF/SGF hybrid laminates.

Sample	*E’* @ 50 °C, [MPa]	*tan delta* [°C]	Effectiveness Coefficient *C*		
			175 °C	200 °C	225 °C
Copolymer	2139	225.5	-	-	-
SDPF-15	2611	229.8	0.76	0.55	0.41
SDPF-30	3448	235.1	0.68	0.50	0.39
SDPF-45	3331	231.1	0.72	0.52	0.42
H(SDPF/SGF)-1	5190	233.7	0.71	0.51	0.43
H(SDPF/SGF)-2	5533	232.8	0.70	0.51	0.43

**Table 5:** TGA summary of SDPF biocomposites and SDPF/SGF hybrid laminates.

Sample	*T*_5_ [°C]	*T*_10_ [°C]	*Y_c_* [%]	*HRI* [°C]	*LOI* [%]
Copolymer	427.6	455.2	66.10	270.21	43.94
SDPF-15	398.0	431.5	61.96	221.48	42.28
SDPF-30	360.5	423.8	56.14	208.84	39.95
SDPF-45	341.6	416.5	50.60	204.25	37.74
H(SDPF/SGF)-1	364.8	438.3	60.89	230.26	41.85
H(SDPF/SGF)-2	365.3	439.9	60.85	230.35	41.84

## Data Availability

Data are contained within the article.

## References

[1] Mallaki M., Fatehi R. (2014). Design of a biomass power plant for burning date palm waste to cogenerate electricity and distilled water. Renew. Energy.

[2] Faiad A., Alsmari M., Ahmed M.M.Z., Bouazizi M.L., Alzahrani B., Alrobei H. (2022). Date Palm Tree Waste Recycling: Treatment and Processing for Potential Engineering Applications. Sustainability.

[3] Yahya S.A., Iqbal T., Omar M.M., Ahmad M. (2021). Techno-Economic Analysis of Fast Pyrolysis of Date Palm Waste for Adoption in Saudi Arabia. Energies.

[4] Chao C.T., Krueger R.R. (2007). The Date Palm (*Phoenix dactylifera* L.): Overview of Biology, Uses, and Cultivation. HortScience.

[5] Mirmehdi S.M., Zeinaly F., Dabbagh F. (2014). Date palm wood flour as filler of linear low-density polyethylene. Compos. Part B Eng..

[6] Al-Khanbashi A., Al-Kaabi K., Hammami A. (2005). Date palm fibers as polymeric matrix reinforcement: Fiber characterization. Polym. Compos..

[7] Maou S., Meghezzi A., Nebbache N., Meftah Y. (2019). Mechanical, morphological, and thermal properties of poly(vinyl chloride)/low-density polyethylene composites filled with date palm leaf fiber. J. Vinyl Addit. Technol..

[8] Saleh A.A., Saleh M.A., Al Haron M.H., Farag M. (2017). Insights into the effect of moisture absorption and fiber content on the mechanical behavior of starch-date-palm fiber composites. Starch Stärke.

[9] Ibrahim H., Farag M., Megahed H., Mehanny S. (2014). Characteristics of starch-based biodegradable composites reinforced with date palm and flax fibers. Carbohydr. Polym..

[10] Sh Al-Otaibi M., Alothman O.Y., Alrashed M.M., Anis A., Naveen J., Jawaid M. (2020). Characterization of Date Palm Fiber-Reinforced Different Polypropylene Matrices. Polymers.

[11] Abu-Sharkh B.F., Hamid H. (2004). Degradation study of date palm fibre/polypropylene composites in natural and artificial weathering: Mechanical and thermal analysis. Polym. Degrad. Stab..

[12] Al-Kaabi K., Al-Khanbashi A., Hammami A. (2005). Date palm fibers as polymeric matrix reinforcement: DPF/polyester composite properties. Polym. Compos..

[13] Kashizadeh R., Esfandeh M., Rezadoust A.M., Sahraeian R. (2019). Physico-mechanical and thermal properties of date palm fiber/phenolic resin composites. Polym. Compos..

[14] Abdal-hay A., Suardana N.P.G., Jung D.Y., Choi K.-S., Lim J.K. (2012). Effect of diameters and alkali treatment on the tensile properties of date palm fiber reinforced epoxy composites. Int. J. Precis. Eng. Manuf..

[15] Gholami M., Ahmadi M.S., Tavanaie M.A., Khajeh Mehrizi M. (2018). Effect of oxygen plasma treatment on tensile strength of date palm fibers and their interfacial adhesion with epoxy matrix. Sci. Eng. Compos. Mater..

[16] Fiore V., Scalici T., Badagliacco D., Enea D., Alaimo G., Valenza A. (2017). Aging resistance of bio-epoxy jute-basalt hybrid composites as novel multilayer structures for cladding. Compos. Struct..

[17] Wang H., Yang L., Wu H. (2020). Study on mechanical and thermomechanical properties of flax/glass fiber hybrid-reinforced epoxy composites. Polym. Compos..

[18] Kumar N., Grewal J.S., Singh A., Mehta V. (2021). A Comparative study of alkali treated date palm fiber based brake friction composites and standard Kevlar-based brake friction composites. Polym. Compos..

[19] Alarifi I.M. (2020). Investigation into the morphological and mechanical properties of date palm fiber-reinforced epoxy structural composites. J. Vinyl Addit. Technol..

[20] Laskoski M., Shepherd A.R., Mahzabeen W., Clarke J.S., Keller T.M., Sorathia U. (2018). Sustainable, fire-resistant phthalonitrile-based glass fiber composites. J. Polym. Sci. Part A Polym. Chem..

[21] Wang A.-r., Dayo A.Q., Zu L.-w., Xu Y.-l., Lv D., Song S., Tang T., Liu W.-b., Wang J., Gao B.-c. (2018). Bio-based phthalonitrile compounds: Synthesis, curing behavior, thermomechanical and thermal properties. React. Funct. Polym..

[22] Chen Y.-P., Dayo A.Q., Zhang H.-Y., Wang A.-r., Wang J., Liu W.-b., Yang Y., Qin Q.-R., Yang Y.-G. (2019). Synthesis of cardanol-based phthalonitrile monomer and its copolymerization with phenol–aniline-based benzoxazine. J. Appl. Polym. Sci..

[23] Ning Y., Li D., Wang M., Jiang L. (2021). Bio-resourced eugenol derived phthalonitrile resin for high temperature composite. J. Appl. Polym. Sci..

[24] Guo H., Lei Y., Zhao X., Yang X., Zhao R., Liu X. (2012). Curing behaviors and properties of novolac/bisphthalonitrile blends. J. Appl. Polym. Sci..

[25] Zhao X., Lei Y., Zhao R., Zhong J., Liu X. (2012). Preparation and properties of halogen-free flame-retarded phthalonitrile-epoxy blends. J. Appl. Polym. Sci..

[26] Xu M., Liu M., Dong S., Liu X. (2013). Design of low temperature self-cured phthalonitrile-based polymers for advanced glass fiber composite laminates. J. Mater. Sci..

[27] Dayo A.Q., Wang A.-r., Derradji M., Kiran S., Zegaoui A., Wang J., Liu W.-b. (2018). Copolymerization of mono and difunctional benzoxazine monomers with bio-based phthalonitrile monomer: Curing behaviour, thermal, and mechanical properties. React. Funct. Polym..

[28] Rajeshkumar G. (2021). Characterization of Surface Modified Phoenix sp. Fibers for Composite Reinforcement. J. Nat. Fibers.

[29] Dayo A.Q., Zegaoui A., Nizamani A.A., Kiran S., Wang J., Derradji M., Cai W.-a., Liu W.-b. (2018). The influence of different chemical treatments on the hemp fiber/polybenzoxazine based green composites: Mechanical, thermal and water absorption properties. Mater. Chem. Phys..

[30] Jones R.M. (1999). Mechanics of Composite Material.

[31] Li X., Tabil L.G., Panigrahi S. (2007). Chemical treatments of natural fiber for use in natural fiber-reinforced composites: A review. J. Polym. Environ..

[32] Rajeshkumar G., Hariharan V., Devnani G.L., Prakash Maran J., Sanjay M.R., Siengchin S., Al-Dhabi N.A., Ponmurugan K. (2021). Cellulose fiber from date palm petioles as potential reinforcement for polymer composites: Physicochemical and structural properties. Polym. Compos..

[33] Lu N., Oza S. (2013). Thermal stability and thermo-mechanical properties of hemp-high density polyethylene composites: Effect of two different chemical modifications. Compos. Part B Eng..

[34] Zegaoui A., Derradji M., Dayo A.Q., Medjahed A., Zhang H.-y., Cai W.-a., Liu W.-b., Ma R.-k., Wang J. (2019). High-performance polymer composites with enhanced mechanical and thermal properties from cyanate ester/benzoxazine resin and short Kevlar/glass hybrid fibers. High Perform. Polym..

[35] Asim M., Paridah M.T., Saba N., Jawaid M., Alothman O.Y., Nasir M., Almutairi Z. (2018). Thermal, physical properties and flammability of silane treated kenaf/pineapple leaf fibres phenolic hybrid composites. Compos. Struct..

[36] Pashaei S., Hosseinzadeh S., Syed A.A. (2017). Studies on coconut shell powder and crysnanoclay incorporated acrylonitrile-butadiene rubber/styrene butadiene rubber (NBR/SBR) green nanocomposites. Polym. Compos..

[37] Fulmali A.O., Sen B., Nayak B.A., Prusty R.K. (2021). Effect of repeated hydrothermal cycling on the durability of glass fiber epoxy. Polym. Compos..

[38] Kiran S., Gorar A.A.K., Wang T., Dayo A.Q., Zhang L.L., Wang J., Shah A.H., Sami S.K., Liu W.B. (2022). Effects of hollow glass microspheres on the polybenzoxazine thermosets: Mechanical, thermal, heat insulation, and morphological properties. J. Appl. Polym. Sci..

[39] Derradji M., Mehelli O., Khiari K., Abdous S., Soudjrari S., Zegaoui A., Ramdani N., Liu W., Al Hassan M. (2022). High performance green composite from vanillin-based benzoxazine containing phthalonitrile and silane surface modified basalt fibers. High Perform. Polym..

[40] Cheng M., Zhong Y., Kureemun U., Cao D., Hu H., Lee H.P., Li S. (2020). Environmental durability of carbon/flax fiber hybrid composites. Compos. Struct..

[41] Ahmad I., Wong P.Y., Abdullah I. (2006). Effects of fiber composition andgraft-copoly(ethylene/maleic anhydride) on thermoplastic natural rubber composites reinforced by aramid fiber. Polym. Compos..

[42] Alshahrani H., Dayo A.Q., Liu W.B. (2022). Development of chopped glass fiber composites with difunctional benzoxazine and bio-based phthalonitrile copolymer: A study of mechanical and thermomechanical properties. J. Appl. Polym. Sci..

[43] Ghouti H.A., Zegaoui A., Derradji M., Cai W.-a., Wang J., Liu W.-b., Dayo A.Q. (2018). Multifunctional hybrid composites with enhanced mechanical and thermal properties based on polybenzoxazine and chopped kevlar/carbon hybrid fibers. Polymers.

[44] Fragassa C., Pavlovic A., Santulli C. (2018). Mechanical and impact characterisation of flax and basalt fibre vinylester composites and their hybrids. Compos. Part B Eng..

[45] Ghori S.W., Rao G.S. (2021). Mechanical and thermal properties of date palm/kenaf fiber-reinforced epoxy hybrid composites. Polym. Compos..

[46] Shanmugam D., Thiruchitrambalam M. (2013). Static and dynamic mechanical properties of alkali treated unidirectional continuous Palmyra Palm Leaf Stalk Fiber/jute fiber reinforced hybrid polyester composites. Mater. Des..

[47] Ornaghi H.L., da Silva H.S.P., Zattera A.J., Amico S.C. (2011). Hybridization effect on the mechanical and dynamic mechanical properties of curaua composites. Mater. Sci. Eng. A.

[48] Pothan L.A., Oommen Z., Thomas S. (2003). Dynamic mechanical analysis of banana fiber reinforced polyester composites. Compos. Sci. Technol..

[49] Devi L.U., Bhagawan S.S., Thomas S. (2010). Dynamic mechanical analysis of pineapple leaf/glass hybrid fiber reinforced polyester composites. Polym. Compos..

[50] Romanzini D., Ornaghi H.L., Amico S.C., Zattera A.J. (2012). Influence of fiber hybridization on the dynamic mechanical properties of glass/ramie fiber-reinforced polyester composites. J. Reinf. Plast. Compos..

[51] Ornaghi H.L., Bolner A.S., Fiorio R., Zattera A.J., Amico S.C. (2010). Mechanical and dynamic mechanical analysis of hybrid composites molded by resin transfer molding. J. Appl. Polym. Sci..

[52] Jawaid M., Abdul Khalil H.P.S., Alattas O.S. (2012). Woven hybrid biocomposites: Dynamic mechanical and thermal properties. Compos. Part A Appl. Sci. Manuf..

[53] Zadeh K.M., Ponnamma D., Al Ali Al-Maadeed M. (2017). Date palm fibre filled recycled ternary polymer blend composites with enhanced flame retardancy. Polym. Test..

[54] Gheith M.H., Aziz M.A., Ghori W., Saba N., Asim M., Jawaid M., Alothman O.Y. (2019). Flexural, thermal and dynamic mechanical properties of date palm fibres reinforced epoxy composites. J. Mater. Res. Technol..

[55] Butler T., Bunton C., Ryou H., Dyatkin B., Weise N., Laskoski M. (2021). Influence of molecular weight on thermal and mechanical properties of bisphenol A-based phthalonitrile resins. J. Appl. Polym. Sci..

[56] Mochane M.J., Mokhena T.C., Mokhothu T.H., Mtibe A., Sadiku E.R., Ray S.S., Ibrahim I.D., Daramola O.O. (2019). Recent progress on natural fiber hybrid composites for advanced applications: A review. Express Polym. Lett..

[57] Van Krevelen D.W., Te Nijenhuis K. (2009). Properties of Polymers.

[58] Zegaoui A., Derradji M., Ma R., Cai W.-a., Medjahed A., Liu W.-b., Dayo A.Q., Wang J. (2018). Silane-modified carbon fibers reinforced cyanate ester/benzoxazine resin composites: Morphological, mechanical and thermal degradation properties. Vacuum.

[59] Zegaoui A., Derradji M., Ghouti H.A., Medjahed A., Zu L.-w., Liu W.-b., Wang J., Dayo A.Q., Liu Y.-g. (2019). Synergetic effects of short carbon/basalt hybrid fibers on the mechanical, thermal and nuclear shielding properties of DCBA/BA-a resin composites. Compos. Commun..

[60] Turku I., Karki T., Puurtinen A. (2018). Durability of wood plastic composites manufactured from recycled plastic. Heliyon.

[61] Moudood A., Rahman A., Öchsner A., Islam M., Francucci G. (2018). Flax fiber and its composites: An overview of water and moisture absorption impact on their performance. J. Reinf. Plast. Compos..

[62] Zhai Z., Feng L., Liu Z., Li G. (2016). Water absorption test for carbon fiber epoxy resin composite based on electrical resistance. Polym. Test..

